# Key informant perspectives on implementing genomic newborn screening: a qualitative study guided by the Action, Actor, Context, Target, Time framework

**DOI:** 10.1038/s41431-024-01650-7

**Published:** 2024-06-21

**Authors:** Erin Tutty, Alison D. Archibald, Lilian Downie, Clara Gaff, Sebastian Lunke, Danya F. Vears, Zornitza Stark, Stephanie Best

**Affiliations:** 1 Murdoch Children’s Research Institute, Melbourne, VIC Australia; 2 Department of Paediatrics, The University of Melbourne, Melbourne, VIC Australia; 3 Victorian Clinical Genetics Service, Melbourne, VIC Australia; 4 Melbourne Genomics Health Alliance, Melbourne, VIC Australia; 5 WEHI, Melbourne, VIC 3052 Australia; 6 Australian Genomics, Melbourne, VIC Australia; 7 Department of Pathology, The University of Melbourne, Melbourne, VIC Australia; 8 Melbourne Law School, University of Melbourne, Melbourne, VIC Australia; 9 Peter MacCallum Cancer Centre, Melbourne, VIC Australia; 10 School of Health Sciences, The University of Melbourne, Melbourne, VIC Australia

**Keywords:** Public health, Genomics

## Abstract

Newborn screening (NBS) programmes are highly successful, trusted, public health interventions. Genomic sequencing offers the opportunity to increase the benefits of NBS by screening infants for a greater number and variety of childhood-onset conditions. This study aimed to describe who needs to do what, when, and for whom to deliver genomic newborn screening (gNBS) and capture perceived implementation barriers and enablers. ‘Key informants’ (individuals involved in the delivery of NBS) were interviewed. The Actor, Action, Context, Time and Target framework guided data collection and analysis. Participants (*N* = 20) identified new Actions required to deliver gNBS (educating healthcare providers, longitudinal psychosocial support), NBS Actions needing modification (obtaining consent) and NBS Actions that could be adopted for gNBS (prompt referral pathways). Obtaining consent in a prenatal Context was a source of some disagreement. The Time to disclose high chance results was raised as a key consideration in gNBS programme design. Genetic counsellors were identified as key Actors in results management, but workforce limitations may be a barrier. Online decision support tools were an enabler to offering gNBS. The implementation of gNBS will require behaviour changes from HCPs delivering NBS. Findings can inform how to deliver gNBS at population-scale.

## Introduction

Newborn bloodspot screening (NBS) aims to improve health outcomes by promptly identifying infants at risk of serious but treatable childhood-onset conditions and facilitating access to treatment. Since its introduction in the 1960s, NBS has become one of the most successful public health programmes worldwide [1]. Advances in technology (i.e., tandem mass spectrometry) saw NBS significantly evolve in the early 2000s, and now, the genomics era offers opportunities for further expansion of NBS [2].

Standard NBS (stdNBS) is typically delivered as a programme, which includes the infrastructure to support the provision of pre-test information, staff to collect and analyse samples, and access to care for infants following a diagnosis [3, 4]. Screening is performed on bloodspot samples which are collected in the first days of life. Parental consent is provided explicitly during sample collection, or implied [5, 6]. Screening panels vary within and across countries but are generally limited to treatable, childhood-onset conditions detectable via biochemical and other functional tests. If an infant receives a ‘high chance’ screening result, the parents are informed, and diagnostic testing is organised. Infants who receive a diagnosis are then referred to specialist physicians for ongoing management of the condition. ‘Low chance’ screening results are typically not disclosed.

To date, genetic testing has been primarily used in stdNBS as a second-tier test to establish a diagnosis following a high chance screening result. Genetic testing may also be offered to determine the carrier status of the infant’s parents and other family members. Several large studies have trialled, or are currently trialling, the use of genomic sequencing as a first-tier test in NBS [2]. This is called genomic newborn screening (gNBS).

gNBS can greatly expand the number and type of conditions able to be screened as part of stdNBS, which would enable early identification of pre-symptomatic infants and facilitate prompt access to treatment. This is particularly important as the number of precision treatments for rare diseases is expanding rapidly. An example of this is spinal muscular atrophy (SMA), for which treatment (gene replacement therapy or medication administered orally or via intrathecal injection) significantly alters the disease progression of pre- or early symptomatic infants. As a result, SMA has now been added to several NBS programmes worldwide [7].

Parents, healthcare professionals (HCPs), and policy makers generally agree that gNBS offers significant benefits to families and to the healthcare system [8]. However, key aspects of the programme design need consideration before gNBS is ready for population-wide implementation [2, 9]. These include ensuring equity of access, developing models to facilitate informed consent, deciding the type of genomic results to report and timing of result return, and ensuring appropriate infrastructure and workforce resources and supports are in place [2]. Care must also be taken to avoid decreasing the high uptake of stdNBS [10]. Guidelines recommend drawing on the strengths of stdNBS programmes and the expertise of those involved in the delivery of stdNBS when considering the implementation of gNBS [3].

### Study aim

This study investigated stakeholder perspectives on implementing gNBS to inform a pilot gNBS study. Specifically, we aimed to:

1. Identify who needs to do what, when, and for whom to deliver gNBS and;
2. Identify perceived barriers and enablers to integrating genomics into NBS.

## Materials and methods

### Context

This study was conducted in the state of Victoria, Australia. In Australia, state/territory health departments are responsible for delivering stdNBS. stdNBS is opt-in in all states/territories, although very few parents decline [6]. The Victorian stdNBS panel currently consists of 27 conditions [4, 11]. Parents provide written consent for stdNBS at the time of the bloodspot sample being collected [6, 12]. Approximately 80,000 infants are born in Victoria each year; 99% have stdNBS and 0.1% are diagnosed with a condition following confirmatory tests [13].

### Study design

This qualitative interview study was guided by the Action, Actor, Context, Target, Time (AACTT) framework [14]. The AACTT framework facilitates detailing of the specifications of the behaviour(s) required for a change and can be applied in a variety of ways when conducting implementation research [14]. Using the AACTT framework, a healthcare behaviour is defined in relation to a specific task (Action) being performed, who (Actor) performs it, where (Context) and when (Time) it is performed, and who it is performed for/with (Target) [14]. The AACTT framework allows identification of both agreement and components where there are differing views on who should do what, where, when and for whom. This information can inform implementation and highlight areas requiring further consideration. Defining behaviours in this way can also inform barriers and enablers to enacting a behaviour change [14].

### Data collection

#### Recruitment

We invited ‘key informants’, i.e., those involved in the delivery of stdNBS across Victoria [15]. Participants included those who invite prospective parents to take up stdNBS, collect bloodspot samples, deliver results, provide care to families in clinical settings or are involved in the delivery of community-based patient support-.

A purposive sampling approach was taken, whereby participants’ role in stdNBS was considered to ensure maximum variation in opinions and perceptions [16, 17]. Potential key informants were identified through study investigator professional networks. We contacted individuals and professional groups via email and invited them to participate and/or distribute recruitment materials to their colleagues. We also used snowball sampling where interview participants recommended other potential individuals to contact [16, 17]. We estimated a sample size of 20–25 participants would be sufficient to address the research aims.

#### Procedure

**Fig. 1:**
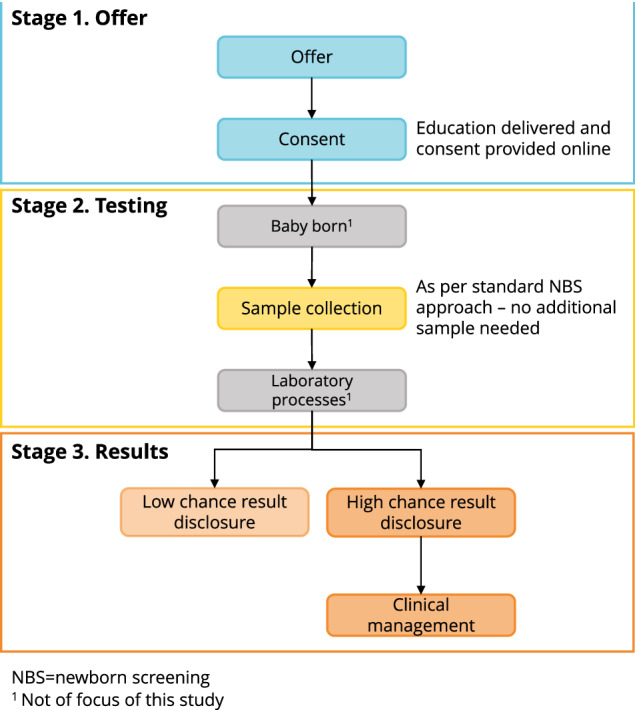
Preliminary genomic newborn screening flowchart.

We developed a gNBS flowchart (Fig. 1), based on the stdNBS process. The flowchart divided gNBS into three stages: (1) offering gNBS, (2) testing and (3) managing results. Laboratory processes were not a focus as policies and regulations purposefully limit variation.

We provided participants with a brief explanation of gNBS and the gNBS flowchart prior to the interview. Participants were asked to consider a model of gNBS where the conditions screened are treatable within the first 5 years of life. Interview questions were focused on the gNBS stage most relevant to the participant’s self-reported role in stdNBS (e.g., midwives were asked about Stage 1 and 2, see Fig. 1). Participants were invited to provide feedback on the proposed flowchart, and, as per the AACTT framework, were prompted to reflect on the Action(s), Actor(s), Context, Time and Target(s) at each gNBS stage. Participants were also asked about barriers and enablers to implementing gNBS.

SB and ET conducted interviews in-person, over telephone or video-call according to participant preference. Interviews were audio-recorded, with verbal consent to participate obtained prior to the interview commencing. Audio-recordings were transcribed and de-identified. Each participant was assigned a participant code based on their professional role.

### Data analysis

We used an abductive approach to data analysis. First, we applied the AACTT framework to the data and deductively coded the transcripts [14]. Barriers, enablers and perceived impacts of gNBS were coded inductively [18, 19]. A sub-set of transcripts were coded and discussed by two authors (SB, ET), before one author (ET) coded the remaining dataset. SB, AA, and ET met regularly to discuss codes, including changes to the proposed gNBS flowchart. Analysis continued until a refined flowchart was complete, with AACTT findings and barriers and enablers defined and described at each stage.

## Results

### Participant characteristics

**Fig. 2:**
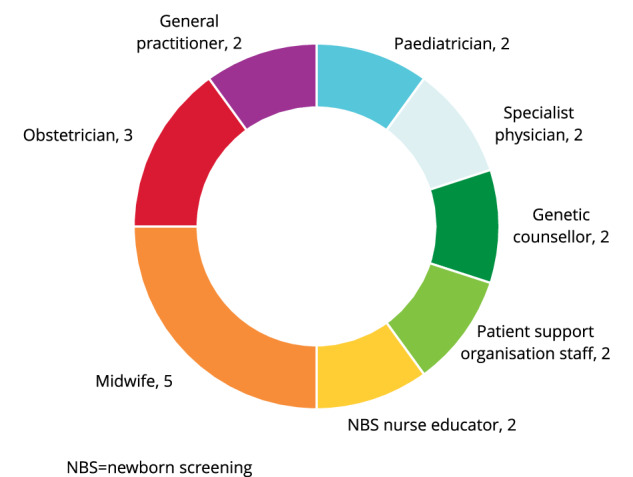
Interview participants (*N* = 20).

Interviews were conducted with 20 participants representing eight professional roles, including 18 HCPs and two staff members from patient support organisations (see Fig. 2). Of the 18 HCP participants, 10 worked in the public healthcare system, two worked in the private healthcare system and three worked publicly and privately. Interviews lasted approximately 33 min each (range, 21–48 min).

### Interview findings

**Fig. 3:**
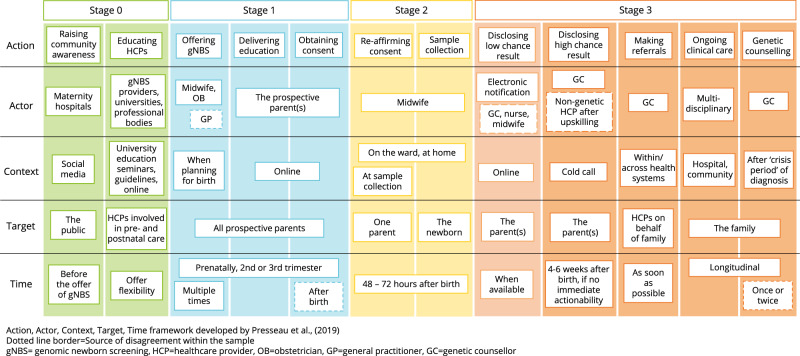
Implementation of genomic newborn screening summarised using the Action, Actor, Context, Target, Time framework.

Participants were mostly supportive of the proposed model of gNBS. Figure 3 outlines the AACTT findings including an additional gNBS stage (Stage 0, awareness and education) and further Actions within stages 1–3. More details are provided below and in Supplementary Table 1.

#### Stage 0—awareness and education

Participants suggested two Actions, ‘raising community awareness’ about gNBS, and ‘educating HCPs’, that could be performed to support the implementation of gNBS. Engagement in gNBS was described as a barrier and an enabler for both Actions. For example, some participants explained that public concern over the use and storage of genomic data may limit interest in gNBS.

*“There’s some hesitation, even with the standard screening about what do they [laboratories] do with their baby’s blood, what is this information for… if you start talking about genomics…there’d be some families that would be quite hesitant about what that meant.”* -Midwife 5

Participants were generally positive about the implementation of gNBS and expressed a willingness to embrace the benefits that genomics offers to NBS. They did not, however, feel prepared to incorporate it into their practice without upskilling first. A range of options for HCP education, including in-services and online training, were suggested.

#### Stage 1—offering genomic newborn screening

Participants identified three Actions involved in the ‘Offer’ stage of gNBS: ‘offering gNBS’, ‘delivering education’ and ‘obtaining consent’. ‘Delivering education’ was added to the gNBS flowchart as an Action after feedback from participants.

Midwives and obstetricians (OBs) were identified as key Actors in offering gNBS. Several participants suggested general practitioners (GPs) may also be well placed to discuss gNBS. GP participants disagreed, however, indicating that they would require upskilling in stdNBS and gNBS first.

The relevance of gNBS to prospective parents (Target) was a key consideration for the Time and Context of the offer. All participants agreed that gNBS should be offered prenatally, and that later in pregnancy, when parents begin preparing for birth, would be most appropriate. Some participants working in obstetrics and midwifery worried that competing clinical priorities and being *“time poor”* (OB2) would deter some HCPs from discussing gNBS. Minimising the clinical impacts of the Actions involved in offering gNBS (e.g., delivering education in an online Context) was recommended. Other participants did not feel that adding genomics into the offer of NBS would have a major impact on practice, however.

*“…this isn’t really changing anything that we’re doing. It’s not extra work for us…It’s just an expansion of something we’re already doing…”* -Midwife 4

Participants felt that they would most likely offer gNBS as an *“add-on”* (OB1) to stdNBS but emphasised that consent should be managed separately.

*“…you can only talk about consent when it’s relevant…knowledge of what [genomic] newborn screening is, that’s a separate education knowledge piece.”* -Support organisation staff member 1

Perspectives on the Time and Context of ‘obtaining consent’ varied. Most were in favour of obtaining consent prenatally but noted that this Action represented one of the more significant differences to stdNBS. Midwives who worked on postnatal wards were more likely to suggest incorporating gNBS into the existing postnatal consent process, feeling that consent would only be relevant to parents after birth. Regardless of the Time and Context, therefore, new processes may need to be developed to support consent for gNBS.

*“Consenting for a genomic screen in that context would need some additional scaffolding. I don’t think it necessarily can’t be embedded in the same process, but I think the workforce will need some serious support.”* -Midwife 1

#### Stage 2—testing

Two Actions were identified at the ‘testing’ stage of gNBS: ‘Re-affirming consent’ and ‘sample collection’. Given that no additional blood sample will be required to perform gNBS, participants did not identify any barriers associated with implementing gNBS at sample collection and agreed that this Action can be performed as per stdNBS processes.

Reminding parents about NBS is an Action typically performed by the midwife at the Time of obtaining consent and collecting the bloodspot. If gNBS consent was to be obtained prenatally, participants suggested this Action could be adapted to ‘re-affirming consent for gNBS’. Re-affirming consent was described as an essential part of gNBS programme design to ensure parents have the option to change their mind prior to blood collection. As described above, obtaining consent prenatally represents a change in practice, and some participants noted that this may be a barrier whilst midwives who collect the bloodspot sample (Actors) adapt to this change.

*“…the challenge anytime you’re changing various established consent processes is… you’ve got someone who’s already been overwhelmed with whatever’s going on in the postnatal ward that day*, [so] *making it as straightforward and clear as possible.”* -Midwife 1

Enablers included Actors being able to *“embrace”* (Midwife 3) the benefits of gNBS, as well as clear communication to HCPs taking the samples, and access to resources and supports.

*“…accessible resources and education…each hospital should have a liaison person…a go-to person that if someone’s unsure about it* [gNBS consent] *they can go to them and they’ve got that maybe slightly higher level of knowledge.”* -Midwife 5

#### Stage 3—managing results

Participants felt that ‘disclosing a low chance result’ was a useful addition to the gNBS process. This is standard practice in the genomics healthcare system, but a change in practice for NBS. The Actions required to manage high chance results were expanded upon from the initial gNBS flowchart. The Actions described were ‘disclosing a high chance result’, ‘making referrals’, ‘ongoing clinical care’ and ‘genetic counselling’. Genetic counsellors (GCs) were identified as key Actors at each stage in results management, although participants noted that limited workforce resources may pose a challenge to population-scale gNBS.

Existing processes for disclosing NBS results (e.g., a *“cold call”* (GC1)) could be adopted for gNBS. GC participants described this Action as *“crisis management”* (GC2) and felt they were best placed to deliver this information. Out of concern for parental bonding, some participants felt that results should be disclosed at 4–6 weeks of life if the result does not have immediate actionability.

*“…baby blues is week one to two… coming out of that… especially if it’s significant news, holding onto it for too much longer* [than 4-6 weeks] *would feel a bit unethical as well.” -*Paediatrician 2

Participants noted that, unlike for conditions already included on stdNBS, established referral pathways and nurse coordinators may not be available for all conditions included on gNBS. As such, this coordination Action would likely be the task of GCs. GCs may also be asked to provide education and support to the non-genetics HCP Actors involved in the ongoing care of infants diagnosed with a condition following gNBS. A lack of genomics knowledge was noted as a barrier to providing ongoing management to patients, particularly amongst GPs.

*“There needs to be more support…and a lot of times when they* [GPs] *come across a syndrome they haven’t come across, they just refer to a paediatrician.”* -GP1

Some non-genetics HCPs explained that genetics and genomics had already begun to be integrated into areas of their practice. Paediatricians noted their profession is familiar with the use of other tests, such as microarrays and exome sequencing, which would support the implementation of gNBS.

*“…we’ve been taught in our training* [about] *microarrays… so then, going to genomics…it makes sense.”* -Paediatrician 1

Beyond medical management, participants also noted the importance of providing families with the option to engage with a patient support organisation. Ongoing funding to patient support organisations would also be necessary.

*“…making sure that the support groups continue to get the funding from the government.”* -Support organisation staff member 2

New models of psychosocial care may also need to be developed to support families who receive a diagnosis for a condition without immediate actionability. GCs explained that the Time and Context of the genetic counselling appointment could vary, depending on the needs of the family. GCs did not, however, consider the role of their profession outside of the *“single session counselling”* (GC2) model usually provided in the stdNBS setting. It was other participants (specialists and support organisation staff) that suggested that longitudinal genetic counselling may be appropriate.

*“We need to create a new paradigm of care which is going to have to involve having these* [psychosocial] *conversations more frequently and finding new ways of supporting these families and reducing harm.”* -Specialist 2

## Discussion

The genomics era offers the potential to build upon the health benefits of NBS, by expanding the number and type of conditions that can be detected and treated early. The design and implementation of a gNBS programme needs to be carefully considered before these health benefits can be realised [2]. Using the AACTT framework to guide data collection and analysis enabled specification of the healthcare behaviours required to effectively deliver a gNBS programme at population-scale in terms of Action(s), Actor(s), Context, Target and Time. Additionally, drawing on the expertise of individuals involved in the delivery of stdNBS enabled an understanding of how to implement gNBS into the relevant healthcare system. Our participants identified aspects of gNBS programme design that could be kept consistent with stdNBS, processes that would require modification, and entirely new elements to consider. Findings have informed the development of a gNBS programme currently under investigation in Australia [20].

Our findings highlight the offer of gNBS, delivering education, and obtaining consent are three distinct and sequential Actions. This differs from the literature on stdNBS, that suggests that the Time and Context of these Actions vary across health services and may occur together [21]. The OBs and antenatal midwives in our study noted that they would discuss stdNBS during pregnancy. Our participants therefore felt that the offer of gNBS could be made alongside discussions about stdNBS, but that consent should be managed separately. This differs to commentary on gNBS suggesting that all Actions related to the offer of gNBS should be performed in a separate Context to stdNBS [8]. This is due to concern that gNBS may reduce uptake in stdNBS [8], especially if stdNBS is first discussed postnatally. Nevertheless, a study in which parents of newborns on the postnatal ward were hypothetically offered gNBS reported high levels of interest in gNBS and no adverse effects on participation in stdNBS [22]. Another study in which gNBS was hypothetically offered found that people had no preference for whether gNBS was offered on the ward alongside stdNBS or during a routine appointment with a paediatrician during the newborn period. This suggests offering gNBS alongside stdNBS may not influence participation in stdNBS or interest in gNBS [23], although evidence from a ‘real-world’ setting is needed to state this conclusively.

Actual uptake of gNBS has been consistently lower than interest in gNBS when offered to parents hypothetically, however [2]. The BabySeq study trialled a model of offering gNBS to parents of healthy newborns on the postnatal ward or to parents of unwell infants in the neonatal intensive care unit [24]. Uptake was less than 10%, with reasons for declining including being uninterested in research and/or gNBS and, particularly for parents of unwell infants, feeling overwhelmed [24]. Similarly, amongst parents of infants with hearing loss offered genome sequencing, uptake of learning about other childhood onset conditions was lowest amongst those with children less than 3 months of age [25]. Whilst adjusting to life with a new baby, deliberating about gNBS may not be a priority [26]. Offering gNBS during pregnancy would provide prospective parents adequate time and resources to support informed decision-making. Parents have likewise expressed a preference for receiving education about gNBS prenatally, specifically within the second and third trimesters [27]. Participants in our study also felt that the most appropriate Time to offer gNBS was during pregnancy. These findings are reflected in our gNBS flowchart.

Midwives have been identified as ideal Actors to provide information and support to prospective parents about stdNBS [6, 13]. Our participants also identified midwives and OBs as Actors in offering gNBS. GP participants did not feel they had a role in offering gNBS, despite being identified as an Actor by other participants. Given time constraints in appointments, participants felt the Action of ‘delivering education’ was best performed using resources prospective parents can engage with in their own Time. Despite parents’ preferences for pre-test information being delivered during pregnancy, recall of information at the time of sample collection is variable [21]. Delivering pre-test information online, with the inclusion of decision support tools, may encourage greater engagement in the content [8]. This may facilitate in-depth deliberation about gNBS [28, 29] and reduce the requirements for HCPs to deliver detailed pre-test information. Online approaches to delivering pre-test information may not be appropriate for, or meet the needs of, all prospective parents. Hybrid approaches, whereby information is provided by the HCP and supplemented by online content, may be appropriate in some contexts. Research is needed to evaluate models of pre-test information delivery at a population-level. Further, our participants noted the importance of reminding parents about gNBS and clarifying consent at the Time of sample collection.

There is consensus that consent for gNBS must be explicitly provided [8]. In the United States, stdNBS is typically mandatory, meaning that a model of explicit, ‘opt-in’ consent for gNBS represents a significant change in practice. Prior to 2011, consent for stdNBS was obtained verbally, or was implied, in Victoria, Australia. Inconsistent approaches to obtaining and documenting parental consent raised concerns about the appropriateness of the method in facilitating informed choice [6]. As such, a written consent process was introduced in 2011. Our participants also supported an ‘opt-in’ approach to gNBS consent, mirroring much of the previous literature [8]. Perspectives on the Context of consent to gNBS also varied; participants in some studies indicate a preference for in-person consent [30], whilst others, including most participants in our study, felt online consent was appropriate [5]. GCs may be considered the ideal Actors to explain the complexities of genomics and clarify patient values during in-person consent processes, as for BabySeq [24], but this approach is limited by workforce constraints. As with pre-test information, an online consent platform would be more feasible if delivering gNBS at population-scale [5, 31, 32]. An online education and consent platform would also allow the Time of consent to occur earlier in the screening process than for stdNBS.

The gNBS consent process was therefore identified as a key Action that would require modification if genomics were to be implemented into NBS. Nisselle et al. [6] investigated how midwives adapted to the 2011 change to NBS consent processes in Victoria, Australia. The authors recommended delivering education about new NBS processes in a variety of Contexts, including offering workshops and online training. Perhaps based on this experience, midwives in our study similarly stressed the importance of disseminating knowledge about changes to consent processes in relation to gNBS.

The Time at which to disclose high chance results has been deliberated. Parents and many HCPs have raised concerns about the impact of a high chance result on parental bonding, particularly for conditions without immediate treatment [33]. Genetic HCPs from the United States felt that the appropriate Time to disclose results about actionable, childhood-onset conditions was at birth, but the group was less unanimous on non-actionable conditions [34]. Although participants in our study were asked to consider gNBS for actionable, childhood-onset conditions only, many held similar concerns about the psychosocial impact of receiving genomic information in the newborn period. Participants suggested disclosing results immediately if action could be taken (as per stdNBS) or 4–6 weeks after birth if the condition was not immediately actionable. Research has also explored the potential to stagger results disclosure based on characteristics of the condition (e.g., age of onset) but the feasibility of this approach at population-scale is yet to be tested. Instead, the focus could be building an appropriate model of longitudinal post-result care for families.

Studies trialling gNBS are still in their infancy, meaning research on the long-term impacts of high chance results on the infants, their families, and health systems is lacking. The BabySeq study was the first to explore the psychosocial implications of gNBS in a sample of 159 parents [35]. Negative psychosocial impacts were minimal up to 10-months post-result, including for the 29 parents who received a monogenic disease risk for their infant [35]. Our participants noted the importance of providing a holistic approach to care that involves the medical management of the infant and the psychosocial wellbeing of the family unit. Longitudinal research is needed to understand how to support families of infants diagnosed after gNBS.

### Limitations

It is possible that the views of all key informants were not captured. Our sampling strategy may have also resulted in an overrepresentation of positive views toward gNBS. Views may change once participants gain experience implementing gNBS and, as such, future research is needed to explore HCP experiences of integrating gNBS into their practice. All participants were professionals involved in the delivery of stdNBS, and research is underway to capture views of prospective parents and members of the public.

## Conclusion

Implementing gNBS represents a change in service delivery requiring HCPs to adapt behaviours or existing stdNBS processes. We drew upon the experience of those who deliver stdNBS to define the behaviours required to implement gNBS. Findings also highlight potential barriers and points of disagreement on Actors, Time and Context of Actions that need attention. The gNBS processes described in this study now require trialling, to assess the feasibility and acceptability of gNBS at population-scale.

## Supplementary information

Implementation of genomic newborn screening summarised using the Actor, Action, Context, Target, Time framework

## Data Availability

Redacted transcripts are available upon reasonable request to the corresponding author.
