# Supplementary material for: Key informant perspectives on implementing genomic newborn screening: a qualitative study guided by the Action, Actor, Context, Target, Time framework
Source: Eur J Hum Genet. 2024 Jun 21;32(12):1599–605. doi: 10.1038/s41431-024-01650-7 (PMC11606939; doi:10.1038/s41431-024-01650-7)
Supplement: Supplementary file 1 — Implementation of genomic newborn screening summarised using the Actor, Action, Context, Target, Time framework [file 41431_2024_1650_MOESM1_ESM.docx]

**Supplementary Table 1. Implementation of genomic newborn screening summarised using the Actor, Action, Context, Target, Time framework**

| **Stage** | **Action** | | **Actor, Context, Target, Time** |
| --- | --- | --- | --- |
| Stage 0: Awareness and education | Raising community awareness | - “There needs to be more community spread messages…” - Midwife 5 | **Actor(s):**   - “…all maternity sites…” - OB2 |
|  |  |  | **Context**   - “…on Instagram or the [maternity hospital] site… ad campaigns and billboards.” - OB2 |
|  |  |  | **Target**   - The “…general population…” - Midwife 5 |
|  |  |  | **Time**   - Before the offer of gNBS: “…definitely flagging it with the general population as something to be aware of…and then when they then fall pregnant that can be raised again.” - Midwife 5 |
|  | Educating HCPs | - “Education directly relevant to healthcare providers” (Midwife 3) is needed to support the implementation of gNBS | **Actor(s):**   - gNBS providers - University education providers - Professional bodies: “...trying to get the RACGP [Royal Australian College of General Practitioners] to implement a little bit of genetic testing into the curriculum…” - GP1 |
|  |  |  | **Context:**   - “…presented in university training or at conferences or regular seminars, or online learning...” - Midwife 4 |
|  |  |  | **Target:**   - “...not just the medical field, like maternal child health nurses, GPs, all the healthcare professionals that intercept with newborn little babies…midwives, obstetricians, paediatricians…” - Paediatrician 1 |
|  |  |  | **Time:**   - Flexibility is important - “...little online course…I find that easy to access and beneficial, I can do it in my own time.” - Midwife 3 |
| Stage 1: Offering gNBS | Offering gNBS | - Performed as per stdNBS: “…we give out the [stdNBS] brochures now in clinic and discuss it with them [prospective parent] and say, ‘have a read, take it home to your partner if they're not in the appointment, come back to us with any questions.’” - Midwife 2 - “I would want to have that [the offer of gNBS] backed up by a website…” - OB3. - gNBS described as an “add-on” (OB1) to stdNBS | **Actor(s)**   - “...midwives are really perfectly positioned...” - Midwife 1 - “...all healthcare practitioners that are involved in the antenatal journey...What I'm talking about is the first and foremost would be the family doctor...” - OB2 - GP participants felt they did not (currently) have a role in offering gNBS: “They [GPs] would just need a lot more [education], to feel a lot more confident about what they were offering…” - GP2 |
|  |  |  | **Context**   - Offering gNBS prenatally would avoid parents making decisions in the “newborn fog” (OB2) after birth: “…if it’s [offered] during pregnancy and antenatal care, that’s not a bad time…during birth, I do know that it’s a bit too overwhelming in terms of how much information is given.” - GP1 - Actors could discuss gNBS in the context of preparing for birth, when the Target is in the “headspace” to “think seriously about would they want the test done.” (OB1) |
|  |  |  | **Target**   - “…it should be definitely offered to everybody…” - OB2 - One or both parents: “I think you can talk to one person…I’m not going to not mention it because the partner couldn’t come. But yes, I think they [the partner] should be involved…” - OB1 |
|  |  |  | **Time**   - “…I would incorporate it at around the 28-week [gestation] mark. Introduce it at least and then say, ‘if you have any other questions, we're going to re-address and talk about birth planning’. Then I start talking to them about it at 36 weeks anyway.” - OB2 - When gNBS becomes relevant to Target: “…that sweet spot of introducing something so parents have enough time. But also not introducing it so far before the baby's born that the parents are still in the space of, ‘well, I've just got to get it out first’…” - Midwife 1 - Multiple times during pregnancy: “We could mention it [gNBS], guide people towards resources, and then maybe bring it up again later.” - OB1 |
|  | Delivering education | - “...delivering clear education to the parents is really, really important.” – NBS nurse educator 2 - “…dot points, short attention span stuff, or a quick video with captions…” - OB2 | **Actor**   - Online, patient-led education: “…they’re actually logging in, and they’re self-educating, instead of just reading a brochure.” – NBS nurse educator 1 - Education delivered by a HCP may be more appropriate for some population groups: “…for our cohort, it would easier for staff members to be conveying that [education] and using translators or interpreters…” - Midwife 5 |
|  |  |  | **Context**   - “…I'm thinking an electronic delivery method of that [education].” - OB2 - “...if it was online, it would need to be in multilingual format and probably quite simplified.” - Midwife 5 |
|  |  |  | **Target**   - Prospective parents: “[after offering gNBS] Then they [the prospective parent] might say, ‘what's that?’ Then you chuck them a link... I'm hoping I could just say...’click on this link, and you can find more about it.’” - OB2 |
|  |  |  | **Time**   - “[online education] gives people a bit more flexibility to do it in their own time…” - Midwife 3 |
|  | Obtaining consent | - Separate to the Actions of offering gNBS and delivering education: “…you can only talk about consent when it’s relevant…knowledge of what [genomic] newborn screening is, that’s a separate education knowledge piece.” - Support organisation staff member 1 - Also managed separately to stdNBS consent: “You'd have to be really clear with separating the consent for the two testings...the consent for the newborn screening and then the genomic screening...” - Midwife 5 | **Actor**   - An online tool that the prospective parent completes: “It will be easy enough to do [consent] on your phone.” - Specialist 1 - Obtained by a HCP, although this would require upskilling: “[It would be] super labour intensive… to have designated midwives at particular facilities who've been upskilled to do the consent process for this add-on...” - Midwife 1 |
|  |  |  | **Context**   - Online (as described above) - Added to the Guthrie card: “…you could just add it to the bottom of the existing card… as a third box to the [stdNBS] consents…” - Midwife 4 |
|  |  |  | **Target**   - “…traditionally people target the mum because she's there with the baby, but that's a bit old fashioned and maybe the dad's got ten extra minutes to sit down and do it [provide consent].” - Specialist 1 |
|  |  |  | **Time**   - “…it’s appropriate to gain consent for testing antenatally.” - OB1 - “…I wouldn’t [obtain consent antenatally] for the simple reason that people are…only fixated on the birth… Anything else that is going to affect the baby after birth, they just dismiss from their thinking…” - Midwife 4 |
| Stage 2: Testing | Baby born | Not a focus of this study | |
|  | Re-affirming consent | - Actors usually remind parents about stdNBS at the time of sample collection and would do the same for gNBS - This Action provides an opportunity to re-affirm consent: “…would you need to consent twice? I think it’s always of value... I think you have to explain that at the time as well.” - Support organisation staff member 1 - …we would need some way to verify that because consent is huge.” - Midwife 3 | **Actor**   - “I would most likely do it [re-affirm consent] myself…” - Midwife 5 |
|  |  |  | **Context**   - “The way I go about it now is that…the women are on the post-natal ward when it comes to that time that the screening is due that I go in and say, ‘has anyone spoken to you about what this [stdNBS] is and what it’s for? Have you had a chance to look at that brochure?’...” - Midwife 3 |
|  |  |  | **Target**   - “…for most people you’d have to re-explain.” - Midwife 4 |
|  |  |  | **Time**   - “Then reiterating when the sample is collected, ‘do you remember you’ve consented for this, do remember what that was for?’” - GC1 |
|  | Sample collection | - “…it [gNBS] wouldn’t change anything.” – Midwife 3 | **Actor**   - “…the midwife looking after the baby, whether that’s on the postnatal ward or the midwife visiting the mother and baby at home…” - Midwife 4 |
|  |  |  | **Context**   - “…on the ward…” – Midwife 3 - “…on a home visit…” – Midwife 3 |
|  |  |  | **Target**   - “…if mum's preference is not to be there, but having someone hold a baby for comfort, we'll do that with another nurse. But most mums are happy to be there, most mums are happy while it's on the breast.” - Midwife 2 |
|  |  |  | **Time**   - “…between 48 and 72 hours [after birth]” - Midwife 3 |
|  | Laboratory processes | Not a focus of this study | |
| Stage 3: Results | Disclosing low chance results | - “…it's good practice with genetic results to be told specifically it was normal.” - Paediatrician 2 | **Actor**   - “Genetic counsellor…the midwife or nurse…” (Paediatrician 2) - “…maternal child health nurse…” (GC2) - “...that's what computers are for. Send them [parents] an email saying, 'if you want screening result… go into this link to the portal for that'.” - Specialist 1 |
|  |  |  | **Context**   - Accessing result online: “…that satisfies the need to tell them [parents] and you avoid having to pay someone, and the difficulties around phone calls and missing people and all that. The downside is you might have people that are not able to access it for various reasons…” - Paediatrician 1 |
|  |  |  | **Target**   - “And sending it [results notification] to both parents versus one parent, that would be something that you'll have to decide.” - Paediatrician 1 |
|  |  |  | **Time**   - “...at the time of a usual check-up…” - GC2 - “...we should just get it to them quickly...” - GC2 |
|  | Disclosing high chance results | - Existing processes for disclosing stdNBS results could be adapted for gNBS - “…crisis management…” (GC2) | **Actor**   - “Genetic counsellors are ideally positioned to be the ones contacting families…”- GC1 - “Currently...the onus is on us as the [specialist team] to disclose that result...it [disclosing gNBS results] should be disease specific...” - Specialist 2 - “… with some upskilling and working with the genetics team I think some general paediatricians could take that on [disclosing increased chance results].” - Paediatrician 1 |
|  |  |  | **Context**   - “…to have this sort of conversation over the phone is anxiety inducing... Face to face conversations are probably better than phone conversations. Obviously, this comes with its own issues of access and equity...” - Specialist 2 - “…we haven’t been able to think of a better way to deliver increased risk results than a cold call.” - GC1 |
|  |  |  | **Target**   - “It is ideal [to disclose the result to both parents at the same time] but it's not always practical.” - Paediatrician 2 |
|  |  |  | **Time**   - “...the 'when' really should be when the result comes. Just like we do with the traditional newborn screening.” - Specialist 2 - “If there is no clinical imperative, I feel like you should leave it for a little while. Maybe for 4-6 weeks is a good time that the parents have bonded, they’ve got some sort of routine.” - GC1 - “...depending upon the condition…would you have the capacity…to say, ‘well, look, this won't present ‘till later on…we have the opportunity to tell the parent a bit later.’ If you did have that, I would say the first three months is pretty tumultuous for parents, so I'd say…around the three month mark.” - Patient advocate 2 |
|  | Making referrals | - “…The next step [after result disclosure] would be to offer a timely in-person long consultation.” - Paediatrician 2 - “It is a lot of case management in the beginning and a lot of logistics” - GC2 - Existing stdNBS pathways could be adapted to suit gNBS: “Just like we do with the traditional newborn screening...if there is an abnormal result you have the conversation with the family and then you get them in the next day to have a more in-depth conversation...that also provides the opportunity to repeat the newborn screen and get the necessary samples off…to look for the relevant biochemical signatures in those fluids.” - Specialist 2 | **Actor**   - “…a team of people with that kind of a [genomics] background to be able to get referrals sent in the right direction to the right people in the first instance…” - GC2 - “There’s not going to be a nurse coordinator for every condition out there so it would fall to genetic counsellors to be identifying those resources and making sure that families are aware of them…” - GC2 |
|  |  |  | **Context**   - Within or across a healthcare system: “...there has to be cross talk between specialties...” - Specialist 2 - “That should be set up before you call the family to say, ‘Are you available?’ [and to] X specialist ‘Have you got time for an appointment the next day?’” - Specialist 1 |
|  |  |  | **Target**   - The Actor is making referrals to HCPs for the family: “…you're going to ring the [clinic] nurse and say, ‘Here's, we've got Baby Jones, when's your team going to be able to see them?’” - Specialist 1 |
|  |  |  | **Time**   - “…within the next day or so.” - GC1 |
|  | Ongoing clinical care | - “A GC is a person who provides the diagnosis and is that person there at that critical acute time, but then they hand over the care of the child to the [specialist] team who are the ones who manage a child with that condition.” - GC1 - “You’re monitoring for symptoms, and you are, in the interim, supporting the family to quell any associated uncertainty.” - Specialist 2 | **Actor**   - “…a full multi-discipline team…” - Support organisation staff member 2 - “…they need to have a paediatrician that is comfortable providing long-term care to them and helping them from a medical point of view, but… also helping them navigating both education and NDIS [National Disability Insurance Scheme] as time goes on.” Paediatrician 2 - “[support groups] have a central role.” - Support organisation staff member 1 - “GPs wouldn’t be seeing these conditions very often at all. If you have a child where they’re really unclear what the condition is, they’re almost always seeing a paediatrician…” - GP2 |
|  |  |  | **Context**   - “…a lot of that [care] can be provided in the early days through the hospital clinics with a plan to eventually move families through to community-based healthcare…” - Paediatrician 2 |
|  |  |  | **Target**   - “…the family and the child…” - Support organisation staff member 2 |
|  |  |  | **Time**   - Longitudinal: “We need to create a new paradigm of care which is going to have to involve having these [psychosocial] conversations more frequently and finding new ways of supporting these families and reducing harm.” – Specialist 2 - “[Time of referral to support group] It has to be at the start…and build that into the system, not kind of add it on afterwards.” - Support organisation staff member 1 |
|  | Genetic counselling | - “… there’s definitely a role for genetic counselling at some stage, particularly before the family has another baby, to understand whether this has come about de novo or whether there’s a reoccurrence risk…” - GC2 | **Actor**   - Genetic counsellors: “Then I would have an appointment with them [the parents] a week or two down the track where…” - GC1 |
|  |  |  | **Context**   - “…an appointment after the crisis period I think, within the next couple of weeks when they’ve got their head around the diagnosis, and they’ve got other questions that are relevant for a genetic counsellor to address…” - GC1 |
|  |  |  | **Target**   - “Usually, it’s a one off or two off, seeing the family for genetic counselling…” – GC2 |
|  |  |  | **Time**   - “a week or two” (GC1) after the diagnosis: “…they’ve had a little bit of time to adapt to whatever the diagnosis is for their child, they’ve gotten to a stage where they can take a step back, think about the genetics, think about what that means for themselves, for future children, for other families, when families are in a headspace where they can consider those other things, that’s when it’s a good time for genetic counselling…” - GC2 |

Action, Actor, Context, Target, Time framework developed by Presseau et al., (2019)

stdNBS=standard newborn screening, gNBS= genomic newborn screening, HCP=healthcare provider, OB=obstetrician, GP=general practitioner, GC=genetic counsellor
